# Iranian Healthcare System Response to Hospital and Intensive Care Bed Requirements During the COVID‐19 Pandemic: A Cross‐Sectional Study

**DOI:** 10.1002/hsr2.70639

**Published:** 2025-04-10

**Authors:** Fazeleh Hemmati, Kasra Jafari, Majid Mirmohammadkhani

**Affiliations:** ^1^ Department of Epidemiology, School of Health Iran University of Medical Sciences Tehran Iran; ^2^ Department of Community Medicine, School of Medicine Semnan University of Medical Sciences Semnan Iran

**Keywords:** COVID‐19, hospital bed capacity, intensive care units, pandemic preparedness

## Abstract

**Background and Aims:**

The COVID‐19 pandemic posed significant challenges to healthcare systems worldwide, including Iran. This article examines the Iranian healthcare system's response to hospital and intensive care unit (ICU) bed requirements during the pandemic. Objectives were to describe the number of total, general, and ICU beds and their change before and during the COVID‐19 outbreak in Iran and to describe the change variations among provinces and in pandemic and post‐pandemic periods.

**Methods:**

A cross‐sectional study design was used to analyze data from the Statistical Centre of Iran. The data set included information on total, general, and ICU beds across different types of healthcare facilities for pre‐ and post‐pandemic periods. We assessed changes in bed counts (both in crude numbers and population‐standardized rates) in the two mentioned periods from March 2015 to 2022.

**Results:**

The study revealed that during the pandemic, there was a notable increase in total, general, and ICU bed counts in Iran. However, when comparing the pre‐ and post‐pandemic periods, ICU beds showed a significantly higher increase (+34.01%) and general beds increased less (+7.47%). The variations in bed count changes among provinces underscored the importance of considering local impacts and resource availability.

**Conclusion:**

The findings demonstrate the adaptability of the Iranian healthcare system to the pandemic. While the system successfully increased ICU bed capacity, there was a shift in resource allocation priorities, with a lesser emphasis on general beds. International comparisons highlight similar strategies employed globally, emphasizing the need for scalable resources during a crisis.

**Abbreviations**
ACU: acute care unit
CCU: coronary care unit
ICU: intensive care unit
IQR: interquartile range
NGO: nongovernmental organizations
NICU: neonatal intensive care unit
NYC: New York City
PACU: post‐anesthesia care unit

## 1 Background

As in the case of COVID‐19, pandemics can cause complicated challenges and serious threats to healthcare systems. Healthcare systems are typically structured and equipped to meet the anticipated demand for health services, particularly for special and professional care such as intensive care. However, they may face significant challenges when confronted with sudden and acute surges in demand, as observed during pandemics. The COVID‐19 pandemic has presented unprecedented challenges to healthcare systems worldwide and the demand for specialized care, including intensive care unit (ICU) beds and critical medical interventions, far exceeded the existing capacity, requiring swift and effective responses to mitigate the spread of the virus and provide adequate care to affected individuals.

COVID‐19 has profoundly impacted Iran's healthcare system, placing immense strain on its infrastructure and resources. The country experienced a significant surge in COVID‐19 cases, leading to a surge in hospitalizations and overwhelming the healthcare system's capacity. The statistics paint a grim picture, with more than 7.6 million confirmed cases and nearly 146,000 fatalities as of January 2025 [1]. ICU beds were in critical demand, with occupancy rates reaching unprecedented levels. A study which analyzed data of more than 1 million hospitalized COVID‐19 patients in Iran over a 26‐month period, reported that 18.26% of these patients admissioned to the ICU wards [2]. From March to August 2021, a total of 270,624 patients with COVID‐19 were admitted to hospitals in Tehran Province, with 31,979 (11.82%) treated in ICU wards [3].

During a crisis such as a pandemic, a resilient healthcare system should be able to effectively adapt in response to dynamic situations while concurrently reducing vulnerabilities across and beyond the system [4]. The global COVID‐19 pandemic has brought to light the inherent limitations of numerous health systems, even those previously regarded as high‐performing and resilient [5]. In the United Kingdom, critical‐care capacity increased from approximately 4000 to 7000 beds, Russia constructed 29 multifunctional medical centers with a total capacity of more than 3000 beds [4], and France provided 4806 new ICU beds (+95% increase) until June 2020 [6].

This study aims to provide a focused evaluation of the Iranian healthcare system's response to the COVID‐19 pandemic by analyzing hospital and intensive care bed capacity before and during the outbreak. While numerous studies have examined Iran's broader response to COVID‐19, no other study have quantitatively assessed how hospital capacity was managed, despite the critical role of bed availability in pandemic preparedness. By examining changes in total, general, and ICU beds, this study offers insights into the healthcare system's resource allocation strategies and adaptability. Given the significant challenges posed by bed shortages during the pandemic, understanding these trends can inform future healthcare planning both in Iran and in other countries with similar healthcare structures.

The primary objective was to describe the number of total, general, and ICU beds and their change before and during the COVID‐19 outbreak in Iran. The secondary objective was to describe the change variations among provinces and in pandemic and post‐pandemic periods.

## 2 Methods and Materials

### 2.1 Study Design

This study utilizes a cross‐sectional design to examine the response of the Iranian healthcare system to hospital and intensive care bed requirements during the COVID‐19 pandemic. This project was found to be in accordance with the ethical principles and the national norms and standards for conducting Medical Research in Iran by the Research Ethics Committee of Semnan University Of Medical Sciences and Health Services (IR.SEMUMS.REC.1402.083).

### 2.2 Data

The primary data source for this study is the Statistical Centre of Iran [7]. The data set includes data on total, general, and ICU beds in the public, private sector, military, nongovernmental organizations (NGOs), tertiary, and research centers' medical care hospitals and centers. The data cover all provinces in Iran and span the period from March 2015 to 2022 encompassing four of the five COVID‐19 peaks in the country. The population of each province was gathered from population forecast reports by the Statistical Center of Iran [8]. Only the the number of approved hospital beds (which the hospital is authorized to maintain and use based on official government permission, and accordingly must have an official license) were included in the analysis.

Our primary data set did not include complete data for Tehran and Yazd Provinces for the latest year, so we used the provincial reports to calculate the bed count for that specific year.

### 2.3 Statistical Analysis

To examine the increase in general and ICU beds during the COVID‐19 outbreak, the absolute differences were calculated for the pre‐pandemic (March 2018 to 2020) and post‐pandemic (March 2020 to 2022) periods. For each period, the difference in bed counts was determined by subtracting the bed count at the beginning of the period from the bed count at the end of the period. Absolute increase values were obtained, representing the actual change in bed numbers over time. Only active bed counts were included in the analysis. An active bed is defined as “a bed that has diagnostic, treatment, support, service, and personnel facilities based on the standards and must be ready for hospitalization and patient care. In other words, it includes occupied or unoccupied hospital beds that can be used for hospitalized patients every day,” according to the Statistical Centre of Iran.

Our data included the number of ICU, total (all types of active hospital beds), and general (all types of beds other than burn, psychiatric, coronary care unit [CCU], and neonatal intensive care unit [NICU] beds) beds.

To account for population differences across provinces, per 100,000 rates of ICU, total, and general beds were calculated. The bed counts were divided by the corresponding population and multiplied by 100,000, resulting in rates that standardized the number of beds per 100,000 individuals. We chose population‐standardized bed rates because it allows for a fair comparison across provinces with different population sizes. Other normalization methods, such as adjusting for healthcare expenditure or hospital density, were not the primary focus of this study, which aimed to assess changes in bed availability in proportion to population needs.

To assess the influence of the COVID‐19 pandemic on bed count changes, the change during the pandemic period was compared to the change in the pre‐pandemic period. The difference between the bed changes during the two periods was calculated for each province. The normality of the data distribution was assessed using the Shapiro−Wilk test, Q−Q plots, histograms, and indices of skewness and kurtosis. To determine the statistical significance of this difference, a two‐sided paired *t*‐test was performed (if the paired *t*‐test's normality assumption were not met we performed a Wilcoxon signed rank test instead). The paired *t*‐test assessed whether there was a significant difference in bed increase between the pandemic and pre‐pandemic periods.

Data were processed using Stata version 11.0. Quantitative variables are expressed as medians and interquartile ranges (IQR). A significance level of *α* = 0.05 was determined as the threshold for statistical significance.

## 3 Results

**Figure 1:**
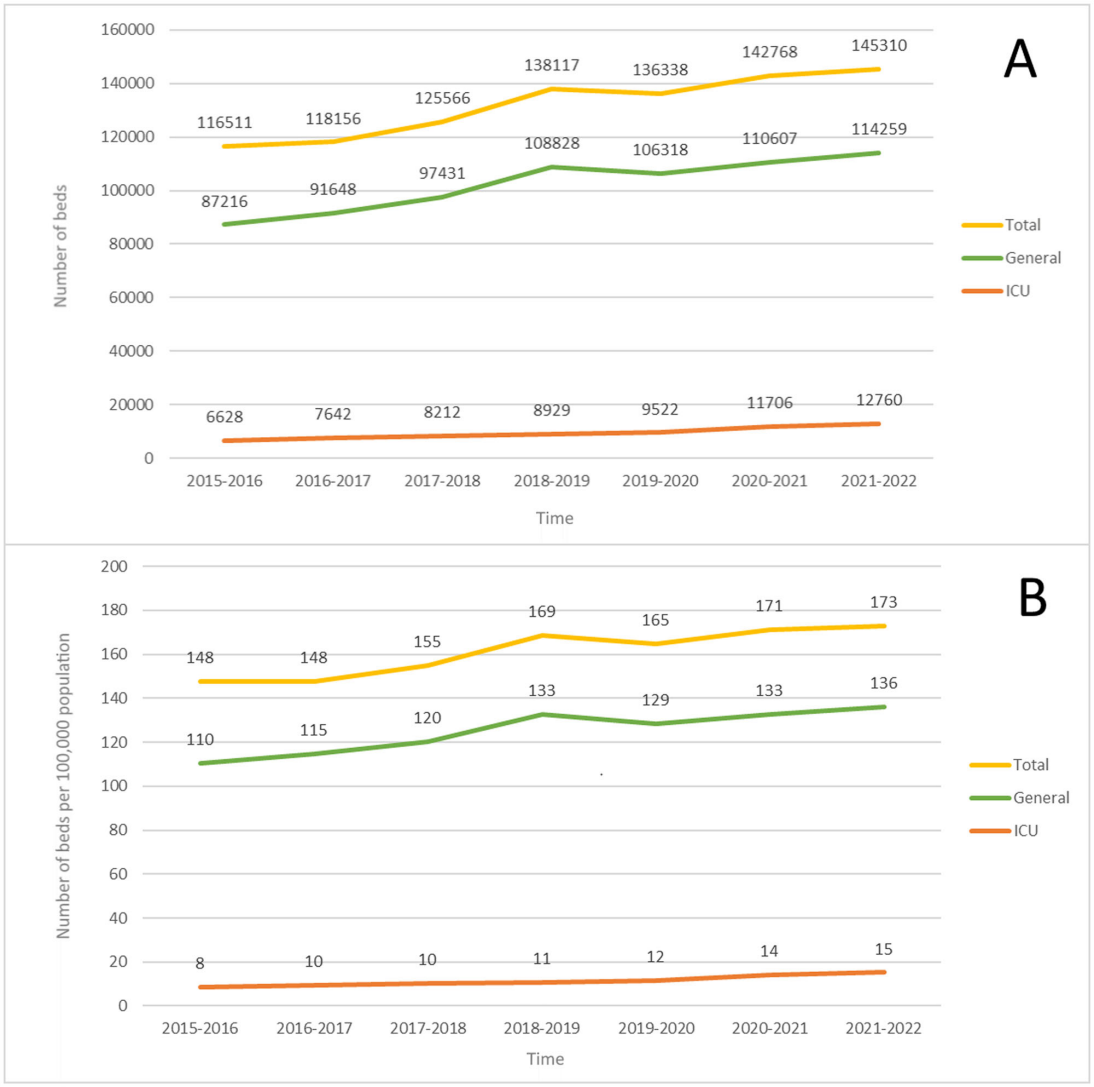
Trend of (A) crude total, general, and ICU bed counts and (B) per 100,000 population bed counts for total, general, and ICU categories changes in Iran 2015−2022.

Figure 1 illustrates the trends in hospital bed capacity in Iran from 2015 to 2022, both in absolute numbers (Panel A) and per 100,000 population (Panel B). The total number of hospital beds has shown a steady increase over the years, rising from 116,511 in 2015−2016 to 145,310 in 2021−2022. This growth is largely driven by an expansion in general hospital beds, which increased from 87,216 to 114,259 over the same period. However, the growth rate of ICU beds, though positive, has been more pronounced in later years, particularly during the COVID‐19 pandemic (2020−2021), where a noticeable jump from 9529 to 11,706 ICU beds is observed.

Panel B, which presents bed counts per 100,000 population, highlights a similar trend but also accounts for population growth. The total bed rate per 100,000 population increased from 148 to 173, while the general bed rate followed a fluctuating trend, stabilizing around 136 beds per 100,000 in 2021–2022. Notably, the ICU bed rate shows a continuous increase, rising from 8 to 15 beds per 100,000 population, reflecting the targeted expansion of ICU capacity, particularly in response to pandemic demands.

Bed counts in the pre‐pandemic and post‐pandemic periods also increased for all of the bed types (Table 1). Median national changes for both periods are available in Table 2. In the pre‐pandemic period, only one province had a decreasing number of total bed counts, but in the post‐pandemic, four provinces decreased their total bed counts. General beds in the pre‐pandemic period decreased in four provinces but decreased in seven provinces in the post‐pandemic, with only South Khorasan decreasing its general beds in both of the periods. However, in the case of ICU bed counts, none of the provinces decreased their bed counts even though five provinces decreased their ICU beds in the pre‐pandemic period.

**Table 1:** Number of pre‐pandemic and post‐pandemic bed counts change for total, general, and ICU categories in Iran 2018−2022 by province.

Province	Total beds		General beds		ICU beds	
	Pre‐pandemic	Post‐pandemic	Pre‐pandemic	Post‐pandemic	Pre‐pandemic	Post‐pandemic
East Azarbaijan	631	775	406	606	30	146
West Azarbaijan	−334	1721	−180	1268	9	246
Ardabil	555	49	506	−13	23	27
Esfahan	780	434	784	247	46	172
Alborz	462	−115	457	−187	−9	105
Ilam	149	129	131	104	7	37
Bushehr	486	98	462	70	21	23
Tehran	462	−2776	27	−1000	385	608
Chaharmahal & Bakhtiyari	192	81	154	59	7	41
South Khorasan	17	41	−10	−8	0	59
Razavi Khorasan	1531	194	1277	−50	148	284
North Khorasan	54	397	46	279	8	42
Khuzestan	129	1479	258	921	14	321
Zanjan	276	183	269	109	−5	66
Semnan	70	93	47	28	24	64
Sistan & Baluchestan	847	157	835	81	−3	58
Fars	2838	722	2300	913	225	132
Qazvin	293	9	223	−11	64	22
Qom	243	111	382	79	64	26
Kordestan	454	122	361	97	11	35
Kerman	875	576	762	435	32	96
Kermanshah	244	225	161	150	33	96
Kohgiluyeh & buyerahmad	473	347	450	310	12	31
Golestan	458	−11	428	−57	15	47
Gilan	215	175	202	92	5	79
Lorestan	139	630	94	539	50	43
Mazandaran	288	327	240	354	36	86
Markazi	36	207	−14	145	9	42
Hormozgan	32	381	35	267	−2	91
Hamedan	41	−11	2	−75	−2	92
Yazd	36	22	−22	3	48	26
Total	10,772	8972	8887	7941	1310	3238

**Table 2:** National median of pre‐pandemic and post‐pandemic bed counts change in Iran 2018−2022.

Bed type	Period	Median	25th percentile	75th percentile
Total beds	Pre‐pandemic	276	70	486
	Post‐pandemic	183	81	434
General beds	Pre‐pandemic	240	46	457
	Post‐pandemic	104	−8	354
ICU beds	Pre‐pandemic	15	7	46
	Post‐pandemic	64	37	105

Table 3 presents the percentage change in hospital bed counts across Iranian provinces during the COVID‐19 pandemic. The data reveal substantial variation in bed expansion efforts. Overall, the total number of hospital beds increased by 6.58%, with general beds rising by 7.47% and ICU beds experiencing the most significant growth at 34.01%.

**Table 3:** Percent change in bed counts in Iran during the pandemic.

Province	Total beds	General beds	ICU beds
East Azarbaijan	10.99	11.46	30.93
West Azarbaijan	45.23	38.73	88.81
Ardabil	1.99	−0.64	24.77
Esfahan	4.9	3.52	31.27
Alborz	−3.64	−7.24	41.5
Ilam	14.14	14.55	67.27
Bushehr	6.72	5.76	26.74
Tehran	−9.35	−4.71	21.82
Chaharmahal & Bakhtiyari	4.75	4.34	69.49
South Khorasan	3.01	−0.77	64.84
Razavi Khorasan	1.82	−0.6	39.23
North Khorasan	34.73	28.62	84
Khuzestan	19.74	15.34	78.68
Zanjan	9.19	6.63	79.52
Semnan	5.79	2.1	48.12
Sistan & Baluchestan	4.29	2.59	35.15
Fars	7.97	13.29	22.56
Qazvin	0.42	−0.62	13.66
Qom	5.17	4.15	21.49
Kordestan	4.4	4.24	28.69
Kerman	10.75	10.27	33.8
Kermanshah	7.06	5.89	44.04
Kohgiluyeh & buyerahmad	24.06	25.75	58.49
Golestan	−0.35	−2.2	25.54
Gilan	4.65	2.95	37.98
Lorestan	24.94	27.19	21.83
Mazandaran	5.87	8.07	18.22
Markazi	9.38	7.96	29.37
Hormozgan	15.99	13.6	73.98
Hamedan	−0.37	−3.08	55.42
Yazd	0.78	0.13	14.61
Total	6.58	7.47	34.01

A key trend is the disproportionate increase in ICU capacity compared to general beds, highlighting the healthcare system's focus on critical care expansion in response to pandemic demands. Provinces such as West Azarbaijan (88.81%), North Khorasan (84%), and Khuzestan (78.68%) experienced some of the highest ICU bed growth rates, likely due to pandemic pressures in these regions. In contrast, general bed expansion was more modest, with some provinces, including Ardabil, South Khorasan, and Golestan, even showing slight declines.

Notably, Tehran and Alborz experienced a reduction in total and general bed counts, with Tehran showing a −9.35% decline in total beds. However, ICU bed expansion in these provinces remained positive, reflecting targeted interventions to enhance critical care capacity.

These trends suggest that while the pandemic accelerated ICU capacity growth nationwide, general hospital infrastructure expansion remained limited in several regions. The uneven distribution of hospital resources may have implications for healthcare equity, underscoring the need for future policies that ensure balanced and sustainable hospital capacity development.

Because the assumptions of a paired *t*‐test were not met, a Wilcoxon signed rank was run to determine whether there was a statistically significant median difference between pre‐ and post‐pandemic period bed count changes. This test determined that there was no statistically significant median increase in total bed counts between the pre‐pandemic (276, IQR: 70−486) and post‐pandemic (183, IQR: 81−434) periods (*z* = 1.724, *p* = 0.085). However, the general bed median increase was significantly lower in the post‐pandemic period (104, IQR: −8 to 354) than in the pre‐pandemic period (240, IQR: 46–457) (*z* = 2.018, *p* = 0.044). For the ICU beds, the median increase in bed counts was significantly higher in the post‐pandemic period (64, IQR: 37−105) than in the pre‐pandemic period (15, IQR: 7−46) (*z* = −3.753, *p* < 0.001).

## 4 Discussion

Iran was one of the countries that was hit hard by the COVID‐19 pandemic, being ranked 18th in total cumulative cases and 12th in total cumulative deaths for the COVID‐19 pandemic [9, 10]. Our findings highlight one of the many ways that Iranian healthcare responded to changes in the demand for healthcare services during the 2 years of the global pandemic. The findings of this study provide valuable insights into the response of the Iranian healthcare system to hospital and intensive care bed requirements during the COVID‐19 pandemic. By examining the changes in bed counts and the variations among provinces, we can better understand the challenges faced and the strategies employed by the Iranian healthcare system to prepare for future challenges.

The observed increase of 6.58% in total beds, 7.47% in general beds, and 34.01% in ICU beds during the pandemic demonstrates the system's adaptability and the ability to scale up resources to meet the heightened demand. These findings align with the resilience and responsiveness expected from a healthcare system during a crisis such as a pandemic. However, compared to the pre‐pandemic change, only ICU beds increased significantly, and the increasing rate of general beds decreased significantly.

Comparing these results with international responses, we can see similarities and differences in the strategies employed in all levels of healthcare systems globally. In New York City, at the peak of the COVID‐19 pandemic, the city's public healthcare system (NYC Health + Hospitals) had an ICU capacity of approximately 300 beds which was increased to over 1000 ICU beds [11]. In the United States, of 3867 hospitals included in a study, 39% increased their ICU beds by 8772 (10.37%) during the early months of the COVID‐19 pandemic [12]. Undertaken measures in South Korea established 3818 more beds for mild to moderate cases [13]. South American countries including Brazil (20%), Chile (212%), Colombia (16%), Ecuador (63%), and Peru (349%) increased their ICU capacity impressively in the first 100 days of the pandemic [14]. From early March 2020 to October 2020, France increased its resuscitation bed capacity by 4200 (72.41%) beds [15]. Additionally, from May to June of the same year in France, 4806 new ICU beds (95% increase) were created from the acute care unit (ACU), post‐anesthesia care unit (PACU), and operating theater, other units or the real build‐up of new ICU beds, respectively and at the peak of the outbreak, 9860, 1982, and 3089 ICU, ACU, and PACU beds were made available, respectively [6].

The variations among provinces in terms of bed count changes are noteworthy. While some provinces demonstrated significant increases in bed counts, others exhibited more modest changes. The increase in ICU beds varied from 13.66% to 88.81% between provinces with a median of 35.15%. This variation could be attributed to differences in the local impact of the pandemic, availability of resources, and regional healthcare infrastructure. It is crucial to consider these variations when developing targeted strategies to ensure equitable access to healthcare resources during pandemics.

It is noteworthy that while the median increase in total bed counts was not different in the pre‐ and post‐pandemic periods, there were significant differences observed in the median increase in general and ICU bed counts. The significantly lower median increase in general beds during the post‐pandemic period suggests a potential shift in resource allocation priorities, with a greater emphasis on ICU bed capacity to cater to severe COVID‐19 cases. These findings highlight the importance of adapting resource allocation strategies based on evolving healthcare needs during a pandemic.

As an important strength, this study provides a comprehensive analysis of Iran's healthcare response to the COVID‐19 pandemic by focusing hospital and ICU bed capacity, a subject that to the best of our knowledge was not assessed or discussed by other studies. Using national data from the Statistical Centre of Iran ensures reliability, while comparing pre‐ and post‐pandemic periods allows for a comparative evaluation of healthcare system resilience over time. Calculating bed rates per 100,000 population allows for standardized comparisons across provinces. Another key strength is the focus on regional disparities in healthcare resource distribution, highlighting the need for equitable policy interventions.

Despite these strengths, the study has some limitations. One key limitation is the incomplete data set for Tehran and Yazd Provinces in the most recent year, requiring reliance on provincial reports for estimates. This could introduce minor biases in the analysis and limit the precision of findings for these regions. Additionally, the study only examines hospital and ICU bed availability without considering other crucial healthcare infrastructure components such as medical equipment, staffing levels, and availability of trained healthcare professionals. While bed expansion is a vital indicator of healthcare preparedness, the actual ability to provide critical care depends on a combination of resources, including ventilators, medications, and skilled personnel. Future research should integrate these elements to offer a more holistic assessment of healthcare system resilience. Another limitation is the study's reliance on yearly data rather than more granular, monthly, or quarterly updates. This limitation prevents a more detailed examination of how the healthcare system responded in real‐time to pandemic surges. Short‐term fluctuations in bed availability and resource allocation strategies may not be fully captured in this analysis. Finally, while the study provides valuable insights into Iran's response, it does not account for patient outcomes or healthcare system strain beyond bed availability. For example, it does not evaluate whether increased ICU capacity was sufficient to meet patient needs or whether shortages in other medical resources affected the quality of care. Future research could incorporate mortality rates, hospitalization durations, and patient flow data to provide a more comprehensive evaluation of the effectiveness of Iran's pandemic response. Most of these limitations stem from data availability constraints, and we had no alternative but to work with the best available sources.

The findings of this study have several implications for the Iranian healthcare system. The significant increase in ICU bed counts and capacity during the pandemic showcases the system's ability to adapt and respond effectively to crises. These insights can inform future preparedness and response efforts, highlighting the importance of prioritizing and enhancing specialized care resources, particularly ICU beds, to ensure optimal patient care during pandemics.

The lessons learned from the Iranian healthcare system's response to the COVID‐19 pandemic can also be applicable in similar contexts globally. The strategies and measures employed to increase bed counts and capacity can provide valuable insights for other countries facing similar challenges. Sharing experiences and best practices can contribute to global efforts in pandemic preparedness and response.

## 5 Conclusion

This study highlights the adaptability of the Iranian healthcare system in responding to the COVID‐19 pandemic, particularly in increasing ICU bed capacity. However, the lower expansion of general hospital beds underscores the need for balanced resource allocation. Strengthening surge capacity, ensuring equitable resource distribution, utilizing real‐time data for informed decision‐making, expanding the healthcare workforce, and developing long‐term pandemic preparedness strategies are crucial for improving resilience. These findings offer insights for middle‐income countries with centralized healthcare systems, particularly those facing resource limitations and uneven healthcare distribution. Countries with similar structures can apply these lessons by adopting flexible ICU expansion strategies, investing in regional healthcare infrastructure, and improving data‐driven decision‐making. Future research should compare different healthcare responses to enhance global preparedness. Integrating these lessons can help healthcare systems improve crisis response and patient outcomes in future public health emergencies.

## Author Contributions

**Fazeleh Hemmati:** conceptualization, data curation, formal analysis, investigation, methodology, resources, validation, visualization, writing – review and editing, writing – original draft. **Kasra Jafari:** conceptualization, data curation, formal analysis, investigation, methodology, validation, visualization, writing – original draft, writing – review and editing. **Majid Mirmohammadkhani:** conceptualization, investigation, project administration, methodology, resources, supervision, validation, writing – original draft, writing – review and editing.

## Ethics Statement

This project was found to be in accordance with the ethical principles and the national norms and standards for conducting Medical Research in Iran by the Research Ethics Committee of Semnan University Of Medical Sciences and Health Services (IR.SEMUMS.REC.1402.083). No human participants were involved in this study. The research committee certificate is available at: https://ethics.research.ac.ir/form/8feqfnryyf0rim83.pdf.

## Consent

The authors have nothing to report.

## Conflicts of Interest

The authors declare no conflicts of interest.

## Transparency Statement

The lead author, Majid Mirmohammadkhani, affirms that this manuscript is an honest, accurate, and transparent account of the study being reported, that no important aspects of the study have been omitted, and that any discrepancies from the study as planned (and if relevant, registered) have been explained.

## Data Availability

The data that support the findings of this study are openly available in Statistical Centre of Iran at: http://amar.org.ir/Portals/0/PropertyAgent/6200/Files/31085/400-99-18.pdf http://amar.org.ir/Portals/0/PropertyAgent/6200/Files/99-99-18.pdf http://amar.org.ir/Portals/0/PropertyAgent/6200/Files/98-99-18.pdf http://amar.org.ir/Portals/0/PropertyAgent/6200/Files/97-99-18.pdf http://amar.org.ir/Portals/0/PropertyAgent/6200/Files/96-99-18.pdf http://amar.org.ir/Portals/0/PropertyAgent/6200/Files/95-99-18.pdf http://amar.org.ir/Portals/0/PropertyAgent/6200/Files/94-99-18.pdf. All data used in this study are available to the public from the website of the Statistical Centre of Iran. Majid Mirmohammadkhani had full access to all of the data in this study and takes complete responsibility for the integrity of the data and the accuracy of the data analysis.
